# Retraction Note: DNA sequence and community structure diversity of multi-year soil fungi in Grape of Xinjiang

**DOI:** 10.1038/s41598-026-56711-2

**Published:** 2026-06-09

**Authors:** Feng Xue, Tong Liu

**Affiliations:** 1 College of Life Sciences, Shihezi University, Shihezi, 832003 China; 2 Guilin Tourism University, Guilin, 541000 China

Retraction of: *Scientific Reports* 10.1038/s41598-021-95854-2, published online 12 August 2021.

The Editors have retracted this Article.

After publication, concerns have been raised about reliability of the references. Specifically, multiple References (3, 6, 7, 19–22, 26, 30, and 32) could not be confirmed to exist, and References 5 and 16 appear to be duplicates. The Authors did not respond to the concerns raised and did not provide the raw data supporting the study.

The Editors have therefore lost confidence in the integrity of this Article.

Feng Xue did not explicitly state whether or not they agree with this retraction. Tong Liu did not respond to correspondence from the Editors about this retraction.

